# Determinants of Metabolic Health Across Body Mass Index Categories in Central Europe: A Comparison Between Swiss and Czech Populations

**DOI:** 10.3389/fpubh.2020.00108

**Published:** 2020-04-08

**Authors:** Sarka Kunzova, Andrea Maugeri, Jose Medina-Inojosa, Francisco Lopez-Jimenez, Manlio Vinciguerra, Pedro Marques-Vidal

**Affiliations:** ^1^International Clinical Research Center, St Anne's University Hospital, Brno, Czechia; ^2^Department of Medical and Surgical Sciences and Advanced Technologies “GF Ingrassia”, University of Catania, Catania, Italy; ^3^Division of Preventive Cardiology, Department of Cardiovascular Medicine, Mayo Clinic, Rochester, MN, United States; ^4^Department of Medicine, Internal Medicine, Lausanne University Hospital, Lausanne, Switzerland

**Keywords:** metabolism, obesity, social factors, behaviors, public health

## Abstract

Comparisons among countries can help to identify opportunities for the reduction of inequalities in cardiometabolic health. The present cross-sectional analysis and meta-analysis aim to address to what extent obesity traits, socioeconomic, and behavioral factors determine poor metabolic health across body mass index (BMI) categories in two urban population-based samples from Central Europe. Data from the CoLaus (~6,000 participants; Lausanne, Switzerland) and the Kardiovize Brno 2030 (~2,000 participants; Brno, Czech Republic) cohorts. For each cohort, logistic regression analyses were performed to identify the main determinants of poor metabolic health overall and stratified by body mass index (BMI) categories. The results of each cohort were then combined in a meta-analysis. We first observed that waist circumference and body fat mass were associated with metabolic health, especially in non-obese individuals. Moreover, increasing age, being male, having low-medium educational level, abdominal obesity, and high body fat mass were the main determinants of the metabolically unhealthy profile in both cohorts. Meta-analysis stratified by BMI categories confirmed the previous results with slight differences across BMI categories. In fact, increasing age and being male were the main determinants of poor metabolic health independent of obesity status. In contrast, low educational level and current smoking were associated with poor metabolic health only in non-obese individuals. In line, public health strategies against obesity and related comorbidities should aim to improve social conditions and to promote healthy lifestyles before the progression of metabolic disorders.

## Introduction

Obesity is a growing plague that imposes devastating health and financial tolls on individuals and society. Obesity is associated with higher mortality rates driven by comorbidities such as type 2 diabetes mellitus, dyslipidemia, hypertension, and certain types of cancer (1). Conversely, a body mass index (BMI) in the normal range is associated with a decreased risk of cardio-metabolic disease and all-cause mortality. However, not all subjects in a certain BMI range have a similar risk. Research on metabolically healthy obesity and metabolically unhealthy obesity (MUO) suggests that for a certain BMI, the risk of cardio-metabolic disease and death varies substantially among subjects (2). The metabolically obese normal weight phenotype, which represents ~10–25% of the normal weight adult population, has been described as a unique subgroup of subjects with metabolic dysregulation similar to obese subjects, despite not being obese according to BMI criteria. The prevalence of metabolically obese normal weight individuals is thus somewhat variable among studies (3). Compared to normal weight and metabolically healthy subjects, metabolically obese normal weight subjects have a 3-fold higher risk of all-cause mortality and/or cardiovascular events (2). Conversely, a large meta-analysis showed that, compared to normal weight and metabolically healthy subjects, subjects with metabolically healthy obesity are not at an increased risk of all-cause mortality and/or cardiovascular events (4). Interestingly, in the meta-analysis the highest risk was found for metabolically obese normal weight (4). To date, it is unclear if metabolically healthy obesity and metabolically obese normal weight status are only transient conditions or permanent phenotypes associated with specific behavioral and/or genetic determinants. Moreover, the prognostic value of metabolically healthy obesity is hotly debated, mainly because it likely shifts gradually toward MUO (3).

In Europe, the rates of cardiovascular disease, obesity and metabolic disorders are substantially higher in groups of lower socioeconomic status, and those inequalities between socio-economic groups tend to increase across a north-south gradient (5). Large variability and absence of such gradient are observed in central, eastern and Baltic European countries (5). Interestingly, the gradient of cardio-metabolic disorders among socioeconomic groups could be partially mediated by several behaviors including diet, physical activity, smoking, and alcohol consumption, which vary enormously among European countries. Thus, comparisons among countries can help to identify opportunities for the reduction of inequalities in health.

To answer these requests, we compared two well-established population-based studies designed to investigate the epidemiology and genetic determinants of cardiovascular and metabolic risk factors: the CoLaus (~6,000 participants; Lausanne, Switzerland) (6) and the Kardiovize Brno 2030 (~2,000 participants; Brno, Czech Republic) (7). Here, our objectives were: (1) to address to what extent obesity traits—especially BMI, waist circumference and body fat mass—determine metabolic health, and (2) to identify the socioeconomic and behavioral determinants associated with unhealthy metabolic profile in the overall population and stratified by BMI categories. To do that, we used data from urban population based samples in two Central European countries, Czech Republic and Switzerland, displaying a similar size population but with a 4-fold difference in gross domestic product (GDP) per capita (8).

## Materials and Methods

### Study Populations

The Kardiovize Brno 2030 study is a population-based prospective cohort study assessing the complex relationships of biological, psychosocial, environmental, and behavioral risk factors with cardiovascular diseases (CVD) outcomes, in a randomly selected ~1% of the urban population of Brno, Czech Republic (7, 9–14). The initial recruitment was completed in 2014, and enrolled 2,160 participants (54.8% women) aged 25–64 years. Follow-up has been planned every 5 years until 2030.

The CoLaus study is a population-based study assessing the clinical, biological, and genetic determinants of cardiovascular disease in the city of Lausanne, Switzerland. Its aims and sampling strategy have been reported previously (6). Recruitment began in June 2003 and ended in May 2006, enrolling 6,733 participants and follow-ups were performed in 2009–2012 and 2014–2017.

Study protocols and baseline examinations were fully described elsewhere (6, 7). Participation rates were 34% in the Kardiovize cohort and 41% in the Colaus cohort, respectively.

### Ethics Approval and Consent to Participate

The baseline study protocol of the Kardiovize Brno 2030 study was approved by the Ethics Committee of St Anne's University Hospital, Brno, Czech Republic (reference 2 G/2012). The institutional Ethics Committee of the University of Lausanne, which afterwards became the Ethics Commission of Canton Vaud (www.cer-vd.ch), approved the baseline CoLaus study (reference 16/03, decisions of 13th January and 10th February 2003), and the approval was renewed for each follow-up. Both studies were performed in agreement with the Helsinki declaration and its former amendments. All participants gave their signed informed consent before entering the study.

### Data Collection and Study Design

In both studies, data were collected by trained interviewers through structured questionnaires. Physical examinations and blood collections after an overnight fasting were performed according to standardized and validated protocols (7). In the current cross-sectional analysis, we used baseline data from participants with complete assessment of socio-economic information and clinical parameters, and no history of CVD.

### Socio-Economic Data

Educational level was categorized as low (primary education or apprenticeship), medium (secondary education), or high (tertiary education). Marital status was categorized into living in couple (married and other relationship) or living alone (single, divorced or widowed). Employment status was categorized into employed (full-time or part-time employment) or unemployed (including retired due to the low prevalence of unemployed). Smoking status was self-reported and defined as current, former or never.

### Clinical Data

In both studies, clinical data, as well as body weight, height and waist circumference (WC) were measured using standard procedures described elsewhere (6, 7). BMI—calculated as weight in kilograms divided by height in meters squared—was used to classify patients as normal weight (BMI < 25 kg/m^2^), overweight (25 ≤ BMI < 30 kg/m^2^), or obese (BMI ≥ 30 kg/m^2^). Due to the low prevalence of underweight participants (BMI <1 8.5 kg/m^2^; 2.2% in Kardiovize cohort, 1.6% in CoLaus cohort), especially of metabolically unhealthy underweight participants in this subgroup, we included only those with BMI ≥ 18.5 kg/m^2^. Abdominal obesity was defined as WC ≥ 102 cm in men and WC ≥ 88 cm in women.

In the Kardiovize study, blood pressure was measured using a mercury sphygmomanometer (Baumanometer, W.A. Baum, Co., Inc., USA). Body fat mass (BFM) was assessed using a direct segmental multi-frequency bioelectrical impedance analysis (InBody 370; BIOSPACE Co., Ltd., Seoul, Korea). Biochemical analyses were performed on fasting blood samples using a Modular SWA P800 analyzer (Roche, Basel, Switzerland). Total cholesterol, triglycerides and glucose were measured by the enzymatic colorimetric method (Roche Diagnostics GmbH, Germany). HDL-cholesterol was measured using the homogeneous method for direct measuring without precipitation (Sekisui Medical, Japan). For triglycerides <4.5 mmol/l, the LDL-cholesterol level was calculated according to the Friedewald equation. For triglycerides >4.5 mmol/l, the LDL-cholesterol was calculated using the homogeneous method for direct measuring (Sekisui Medical, Japan).

In the Colaus study, blood pressure was measured using an Omron® HEM-907 automated oscillometric sphygmomanometer after at least a 10-min rest in a seated position, and the average of the last two measurements was used. BFM was assessed by electrical bioimpedance using the Bodystat® 1500 body mass analyzer (Bodystat Ltd, Isle of Man, England). In a subset of 794 women who also had their body composition assessed using DEXA, the correlation for BFM estimated by bioimpedance and DEXA was 0.852 (*p* < 0.001), with only a slight overestimation by bioimpedance relative to DEXA (+0.9 kg). Biological analyses were performed on a Modular P apparatus (Roche Diagnostics, Basel, Switzerland). The following analytical procedures (with maximum inter and intra-batch CVs) were used: total cholesterol by CHOD-PAP (1.6–1.7%); HDL-cholesterol by CHOD-PAP + PEG + cyclodextrin (3.6–0.9%); triglycerides by GPO-PAP (2.9–1.5%); glucose by glucose dehydrogenase (2.1–1.0%).

### Definition of Metabolic Health

According to the International Diabetes Federation (15), we defined metabolic unhealthy individuals those who presented at least one of the following criteria: (i) systolic/diastolic blood pressure ≥130/85 mm Hg or use of antihypertensive drug; (ii) triglycerides level ≥150 mg/dl; (iii) HDL-cholesterol level < 40 mg/dl in men or < 50 mg/dl in women or use of lipid-lowering drugs; (iv) glucose level ≥100 mg/dl or use of antidiabetic drug. This partially differed from the definition of metabolic syndrome because it did not depend on abdominal obesity. Based on this definition, participants were classified as metabolically healthy or metabolically unhealthy in the whole cohorts and within normal weight, overweight, and obese subgroups.

### Inclusion and Exclusion Criteria

In this cross-sectional analysis, we used data from Kardiovize and CoLaus members with complete assessment of anthropometric and metabolic parameters, and no history of CVD.

### Statistical Analysis

Statistical analyses were performed separately for the Kardiovize and Colaus cohorts using the SPSS software (version 25.0, SPSS, Chicago, IL). The Kolmogorov-Smirnov test was used to test the normality of continuous variables prior to further analyses. Descriptive results are expressed as number of participants (percentage) for categorical variables, or as median (interquartile range, IQR) for continuous variables with skewed distribution. Bivariate analyses were performed using Chi-square test for bivariate or categorical variable, and Mann-Whitney *U*-test or Kruskal–Wallis test for continuous variables. We first evaluated whether anthropometric measures were associated with metabolically unhealthy profile in the whole cohorts and stratified by BMI categories. To do that, we applied different logistic regression models including BMI, waist circumference or body fat mass as independent variable and adjusting for social and behavioral factors that were significantly associated with the unhealthy metabolic profile in at least one cohort (i.e., age, gender, educational level, marital status, employment status, and smoking status). Next, we investigated the main social and behavioral factors associated with metabolically unhealthy profile in the whole cohorts and stratified by BMI categories. In this case, we used logistic regression models including social and behavioral factors that were significantly associated with the unhealthy metabolic profile in at least one cohort (i.e., age, gender, educational level, marital status, employment status, and smoking status). We also tested for interaction between BMI and social or behavioral factors to understand if the estimates for the same exposure differed across BMI categories. Results were expressed as Odds ratio (OR) and 95% confidence interval (CI). All statistical tests were two-sided, and *p* < 0.05 were considered statistically significant.

### Meta-Analysis

For each association between behavioral or social factors and metabolic health, we meta-analyzed the adjusted ORs obtained from each cohort using the Review Manager software (Version 5.3.1). The obtained pooled ORs were reported for the overall population or stratified by BMI categories. For each meta-analysis, heterogeneity across studies was measured using the *Q*-test and *p* < 0.1 was considered statistically significant (16). According to heterogeneity across studies, we used the fixed-effects model (Mantel–Haenszel method) when heterogeneity was negligible or the random-effects models (DerSimonian-Laird method) when heterogeneity was significant. The significance of pooled ORs was determined by the *Z*-test. Except for the *Q*-test, *p* < 0.05 was considered statistically significant, and all tests were 2-sided.

## Results

### Selection and Characteristics of Included Participants

**Figure 1:**
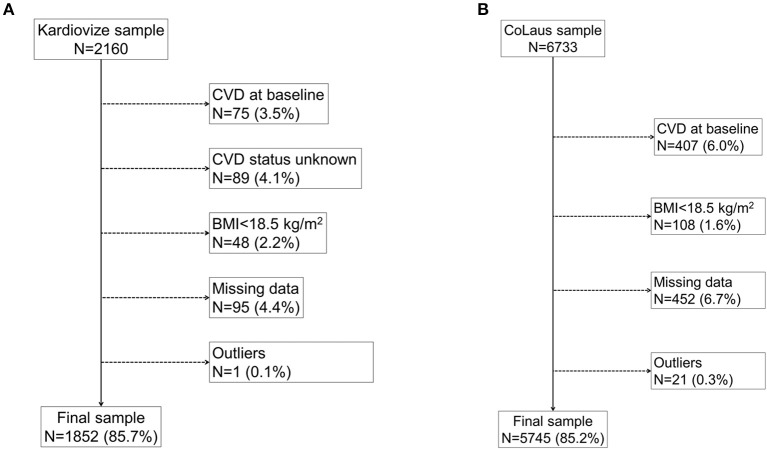
Fluxogram of participants' inclusion in the current analysis. The fluxogram displays the flow of participants and reasons for exclusion in the current analysis for the Kardiovize **(A)** and Colaus **(B)** cohorts.

Figure 1 displays the flowchart of participants' inclusion in the current analysis. From the initial 2,160 participants of the Kardiovize cohort, 308 were excluded because of missing or outlier data (*n* = 185), BMI < 18.5 kg/m^2^ (*n* = 48) or history of CVD (*n* = 75). From the initial 6,733 participants of the Colaus cohort, 988 were excluded because of missing or outlier data (*n* = 473), BMI < 18.5 kg/m^2^ (*n* = 108) or history of CVD (*n* = 407). The characteristics of included participants according to BMI categories are summarized in Table 1. In both cohorts, overweight and obese participants were older, more frequently men, less educated, and less frequently employed or living alone than their normal weight counterparts. The proportion of never smokers decreased with increasing BMI among the Kardiovize participants, while it increased in the Colaus cohort. Similarly, the proportion of current smokers increased with increasing BMI in the Kardiovize while it decreased in the CoLaus cohort.

**Table 1:** Characteristics of participants according to BMI categories.

**Characteristics^**a**^**	**Kardiovize (*****n*** **=** **1,852)**				**Colaus (*****n*** **=** **5,745)**			
	**Normal**	**Overweight**	**Obese**	***P*-value**	**Normal**	**Overweight**	**Obese**	***P*-value**
Sample size	887	631	334		2,756	2,126	863	
Men	35.7% (317)	59.3% (374)	46.1% (154)	<0.001	37.7% (1,038)	58.4% (1,241)	50.1% (432)	<0.001
Age, years	43.0 (18.0)	49.0 (18.0)	53.5 (14.0)	<0.001	49.1 (17.0)	53.3 (17.0)	55.2 (16.0)	<0.001
Marital status				0.002				<0.001
Living alone	41.8% (371)	33.0% (208)	36.9% (123)		43.7% (1,205)	38.6% (822)	36.8% (318)	
Living in couple	58.2% (516)	67.0% (423)	63.1% (211)		56.3% (1,551)	61.4% (1,304)	63.2% (545)	
Educational level				<0.001				<0.001
High	48.6% (431)	39.9% (252)	29.0% (97)		24.3% (670)	16.7% (355)	9.2% (79)	
Medium	37.1% (329)	38.5% (243)	41.6% (139)		26.9% (740)	22.6% (480)	18.5% (160)	
Low	14.4% (127)	21.6% (136)	29.3% (98)		48.8% (1,346)	60.7% (1,291)	72.4% (625)	
Employment status				<0.001				<0.001
Unemployed	15.1% (134)	16.3% (99)	25.2% (104)		25.6% (706)	31.9% (678)	42.3% (365)	
Employed	84.9% (753)	83.7% (509)	74.8% (250)		74.4% (2,050)	68.1% (1,448)	57.7% (498)	
Smoking status				0.006				<0.001
Never	55.1% (489)	47.1% (297)	48.5% (162)		40.7% (1,122)	40.0% (849)	44.5% (384)	
Former	18.3% (162)	25.4% (160)	23.4% (78)		29.0% (798)	34.9% (741)	34.9% (301)	
Current	26.6% (236)	27.5% (174)	28.1% (94)		30.3% (836)	25.1% (534)	20.6% (178)	
Abdominal obesity	2.6% (23)	42.3% (266)	97.0% (324)	<0.001	4.2% (116)	37.2% (790)	91.8% (792)	<0.001
BMI, kg/m^2^	22.5 (2.8)	27.1 (2.4)	33.8 (4.3)	<0.001	22.6 (2.7)	27.1 (2.3)	32.5 (3.9)	<0.001
Waist circumference, cm	79.0 (11.0)	94.0 (11.0)	107.0 (14.0)	<0.001	80.0 (12.0)	94.0 (11.0)	106.0 (13.0)	<0.001
Body fat mass, %	21.6 (10.2)	26.6 (12.6)	38.2 (13.4)	<0.001	26.0 (12.0)	28.0 (13.7)	37.8 (15.0)	<0.001
SBP, mm Hg	113.0 (17.0)	120.5 (18.5)	127.0 (19.0)	<0.001	121.0 (20.5)	129.8 (21.5)	134.5 (24.0)	<0.001
DBP, mm Hg	76.5 (12.0)	81.5 (12.5)	84.0 (11.0)	<0.001	76.0 (13.0)	80.5 (13.5)	84.0 (13.0)	<0.001
Glucose, mmol/L	4.7 (0.70)	5.0 (0.7)	5.2 (0.8)	<0.001	5.2 (0.6)	5.5 (0.8)	5.7 (1.1)	<0.001
Total cholesterol, mmol/L	5.0 (1.3)	5.3 (1.3)	5.2 (1.4)	<0.001	5.4 (1.3)	5.7 (1.4)	5.6 (1.4)	<0.001
Triglycerides, mmol/L	0.8 (0.5)	1.3 (0.9)	1.4 (0.8)	<0.001	0.9 (0.6)	1.3 (0.9)	1.5 (1.1)	<0.001
HDL cholesterol, mmol/L	1.6 (0.5)	1.4 (0.5)	1.3 (0.4)	<0.001	1.8 (0.6)	1.5 (0.5)	1.4 (0.5)	<0.001
LDL cholesterol, mmol/L	2.89 (1.15)	3.25 (1.18)	3.23 (1.18)	<0.001	3.1 (1.1)	3.5 (1.2)	3.4 (1.1)	<0.001
Metabolic health				<0.001				<0.001
Healthy	89.9% (797)	63.5% (401)	41.3% (138)		78.5% (2,163)	49.1% (1,044)	28.5% (246)	
Unhealthy	10.1% (90)	36.5% (230)	58.7% (196)		21.5% (593)	50.9% (1,082)	71.5% (617)	
Diagnosis of type 1 diabetes	1.0% (9)	0.5% (3)	0.0% (0)	0.115	0.2% (5)	0.2% (4)	0.2% (2)	0.956
Diagnosis of type 2 diabetes	0.8% (7)	3.5% (22)	8.7% (29)	<0.001	1.2% (34)	3.4% (73)	12.9% (111)	<0.001
Oral antidiabetic treatment	0.2% (2)	3.5% (22)	7.2% (24)	<0.001	0.8% (21)	2.5% (53)	10.2% (88)	<0.001
Insulin treatment	1.1% (10)	0.5% (3)	0.9% (3)	0.399	0.4% (11)	0.4% (8)	1.6% (14)	<0.001
Diagnosis of hypertension	16.6% (146)	33.7% (210)	62.8% (209)	<0.001	14.3% (393)	29.8% (634)	49.0% (423)	<0.001
Antihypertensive drug treatment	9.2% (82)	21.6% (136)	49.1% (164)	<0.001	7.8% (215)	19.3% (411)	36.7% (317)	<0.001
Diagnosis of high cholesterol	20.4% (176)	36.4% (220)	38.0% (124)	<0.001	14.9% (412)	26.8% (569)	33.7% (291)	<0.001
Hypolipidemic drug treatment	2.8% (25)	9.4% (59)	14.4% (48)	<0.001	6.1% (170)	12.6% (268)	18.0% (156)	<0.001

*BMI, body mass index; SBP, systolic blood pressure; DBP, diastolic blood pressure; HDL, high-density lipoprotein; LDL, low-density lipoprotein. ^a^Results are reported as median (interquartile range) or percentage. Statistical analysis using Chi-square test for bivariate or categorical variable, and Kruskal–Wallis test for continuous variables*.

In both cohorts, all anthropometric and clinical parameters increased from normal weight to obese participants, except of HDL-cholesterol, which decreased. As expected, the prevalence of abdominal obesity, diagnosed and treated hypertension, type 2 diabetes and hypercholesterolemia, were higher in the overweight and obese subgroups. The proportion of metabolically unhealthy participants increased across BMI categories, ranging from 10.1 to 58.7% in the Kardiovize cohort, and from 21.5 to 71.5% in the Colaus cohort.

### Anthropometric Measures and Metabolic Health

The characteristics of participants according to their metabolic status are reported in the Supplementary Material. In both cohorts, metabolically unhealthy participants were older, more frequently men and smokers, and less educated and employed than their metabolically healthy counterparts. In the Colaus cohort, metabolically unhealthy participants were also less likely to live alone. In both cohorts, metabolically unhealthy participants exhibited higher BMI, waist circumference and body fat mass, which resulted in a higher prevalence of abdominal obesity compared with those who were metabolically healthy. Compared with the latter, metabolically unhealthy participants also exhibited higher levels for most clinical parameters, except for beneficial HDL-cholesterol levels, which were lower. The prevalence of diagnosed or treated hypertension, diabetes, and hypercholesterolemia was higher in metabolically unhealthy participants than in metabolically healthy participants.

Notably, logistic regression analysis showed that BMI, waist circumference and body fat mass were positively associated with metabolically unhealthy profile in both cohorts, after adjusting for age, gender, educational level, marital status, employment status, and smoking status (Table 2). Moreover, stratified analysis confirmed the association of waist circumference and body fat mass with metabolic status among normal weight and overweight individuals. By contrast, no association of waist circumference or body fat mass with metabolically unhealthy profile was evident among obese participants (Table 2).

**Table 2:** Association of anthropometric measures with metabolically unhealthy status.

**Characteristics**	**Kardiovize**			**CoLaus**		
	**OR**	**95% CI**	***p*-value**	**OR**	**95% CI**	***p*-value**
**Overall**						
BMI, kg/m^2a^	1.17	(1.14–1.20)	<0.001	1.17	(1.14–1.20)	<0.001
Waist circumference, cm^a^	1.08	(1.06–1.11)	<0.001	1.08	(1.06–1.09)	<0.001
Body fat mass, %^a^	1.08	(1.06–1.10)	<0.001	1.08	(1.06–1.09)	<0.001
**Normal weight**						
Waist circumference, cm^a^	1.08	(1.04–1.14)	0.001	1.05	(1.03–1.08)	<0.001
Body fat mass, %^a^	1.09	(1.04–1.14)	0.001	1.05	(1.03–1.08)	<0.001
**Overweight**						
Waist circumference, cm^a^	1.05	(1.01–1.09)	0.027	1.03	(1.01–1.06)	0.004
Body fat mass, %^a^	1.05	(1.01–1.09)	0.024	1.02	(1.01–1.04)	0.004
**Obese**						
Waist circumference, cm^a^	1.03	(0.98–1.07)	0.217	1.02	(0.98–1.05)	0.354
Body fat mass, %^a^	1.03	(0.99–1.08)	0.194	1.02	(0.98–1.05)	0.342

a *Per one unit increase; BMI, body mass index. Results are expressed as multivariable-adjusted odds ratio (OR) and 95% confidence interval (CI). Statistical analysis was performed using logistic regression models including BMI, waist circumference or body fat mass as independent variable and adjusting for age, gender, educational level, marital status, employment status, and smoking status*.

### Social and Behavioral Determinants of Metabolic Health Across BMI Categories

We next evaluated social and behavioral factors associated with metabolic health in the overall population. Notably, logistic regression analysis demonstrated that increasing age, being male, and having low-medium educational level were the main determinants of the metabolically unhealthy profile in both cohorts. Further, we observed significant associations with unemployed status in the Kardiovize cohort, and with current smoking in the Colaus cohort (Table 3).

**Table 3:** Multivariable associations of social and behavioral factors with metabolically unhealthy status.

**Characteristics**	**Kardiovize**			**CoLaus**		
	**OR**	**95% CI**	***p*-value**	**OR**	**95% CI**	***p*-value**
**Overall**						
Age^a^	1.03	(1.04–1.06)	<0.001	1.04	(1.03–1.05)	<0.001
Man vs. woman	6.55	(4.61–9.31)	<0.001	8.51	(7.03–10.30)	<0.001
Educational level			0.003^b^			0.001^b^
High	1	(ref.)		1	(ref.)	
Medium	1.61	(1.21–2.13)	0.001	1.14	(0.94–1.38)	0.192
Low	1.12	(0.79–1.57)	0.534	1.43	(1.21–1.70)	<0.001
Living alone vs. living in couple	1.06	(0.82–1.37)	0.670	1.02	(0.90–1.16)	0.756
Unemployed vs. employed	1.47	(1.04–2.06)	0.028	0.99	(0.85–1.17)	0.940
Smoking status			0.122^b^			0.002^b^
Never	1	(ref.)		1	(ref.)	
Former	0.93	(0.68–1.27)	0.641	1.10	(0.96–1.28)	0.181
Current	1.28	(0.96–1.71)	0.090	1.32	(1.13–1.54)	<0.001
**Normal weight**						
Age^a^	1.07	(1.04–1.10)	<0.001	1.06	(1.04–1.07)	<0.001
Man vs. woman	4.57	(2.26–9.26)	<0.001	6.31	(4.60–8.65)	<0.001
Educational level			0.013^b^			<0.001^b^
High	1	(ref.)		1	(ref.)	
Medium	2.31	(1.32–4.05)	0.003	1.22	(0.90–1.66)	0.194
Low	1.90	(0.95–3.82)	0.072	1.75	(1.34–2.28)	<0.001
Living alone vs. living in couple	1.36	(0.83–2.23)	0.230	1.03	(0.84–1.26)	0.811
Unemployed vs. employed	1.12	(0.58–2.17)	0.738	1.01	(0.77–1.31)	0.968
Smoking status			0.854^b^			0.008^b^
Never	1	(ref.)		1	(ref.)	
Former	0.90	(0.45–1.78)	0.818	1.06	(0.83–1.34)	0.630
Current	1.12	(0.64–1.98)	0.736	1.44	(1.13–1.83)	0.003
**Overweight**						
Age^a^	1.04	(1.02–1.06)	<0.001	1.04	(1.03–1.05)	<0.001
Man vs. woman	5.26	(2.80–9.90)	<0.001	4.24	(3.02 - 5.94)	<0.001
Educational level			0.518^b^			0.875^b^
High	1	(ref.)		1	(ref.)	
Medium	1.27	(0.83–1.92)	0.269	1.05	(0.78–1.42)	0.753
Low	1.21	(0.74–1.99)	0.448	1.05	(0.87–−1.35)	0.682
Living alone vs. living in couple	1.01	(0.69–1.50)	0.948	0.98	(0.80–1.15)	0.889
Unemployed vs. employed	1.53	(0.89–2.61)	0.121	0.88	(0.70–1.12)	0.294
Smoking status			0.005^b^			0.035^b^
Never	1	(ref.)		1	(ref.)	
Former	0.76	(0.48–1.20)	0.239	1.16	(0.94–1.43)	0.178
Current	1.69	(1.11–2.57)	0.015	1.36	(1.08–1.72)	0.010
**Obesity**						
Age^a^	1.03	(1.01–1.06)	0.024	1.06	(1.04–1.08)	<0.001
Man vs. woman	3.30	(1.57–6.93)	0.002	3.12	(1.81–5.40)	<0.001
Educational level			0.022^b^			0.400^b^
High	1	(ref.)		1	(ref.)	
Medium	1.87	(1.03–3.37)	0.039	0.83	(0.45–1.52)	0.545
Low	0.86	(0.45–1.65)	0.651	1.09	(0.62–1.91)	0.763
Living alone vs. living in couple	1.03	(0.62–1.70)	0.910	1.31	(0.94–1.83)	0.117
Unemployed vs. employed	1.80	(0.94–3.43)	0.076	1.25	(0.86–1.81)	0.248
Smoking status			0.693^b^			0.392^b^
Never	1	(ref.)		1	(ref.)	
Former	1.22	(0.65–2.28)	0.543	1.10	(0.76–1.59)	0.620
Current	0.91	(0.51–1.62)	0.739	1.35	(0.88–2.08)	0.171

a Per one unit increase;

b *p-value for trend. BMI, body mass index. Results are expressed as multivariable-adjusted odds ratio (OR) and 95% confidence interval (CI). Statistical analysis was performed using logistic regression models including all the variables in the table (i.e., age, gender, educational level, marital status, employment status, and smoking status)*.

We also observed a significant interaction between educational level and BMI categories on the relationship with metabolic health (*p* = 0.008). Although no interactions between BMI and other social and behavioral factors were evident (*p* > 0.05), we performed stratified analysis by BMI categories (bivariate analyses are showed in Supplementary Material).

Logistic regression analysis among normal weight individuals demonstrated that increasing age, being male, and having a low-medium educational level were the main determinants of a metabolically unhealthy profile in both cohorts, while current smoking maintained a significant association with a metabolically unhealthy profile in the CoLaus cohort (Table 3). Among overweight individuals, increasing age, being male, and current smoking were the main social and behavioral determinants of the metabolically unhealthy profile in both cohorts (Table 3). Among obese subjects, increasing age and being male were the main determinants of a metabolically unhealthy profile in both cohorts, while having a medium educational level was significantly associated with unhealthy metabolic status only in the Kardiovize cohort (Table 3).

### Meta-Analysis of the Associations of Social and Behavioral Factors With Metabolic Health

**Figure 2:**
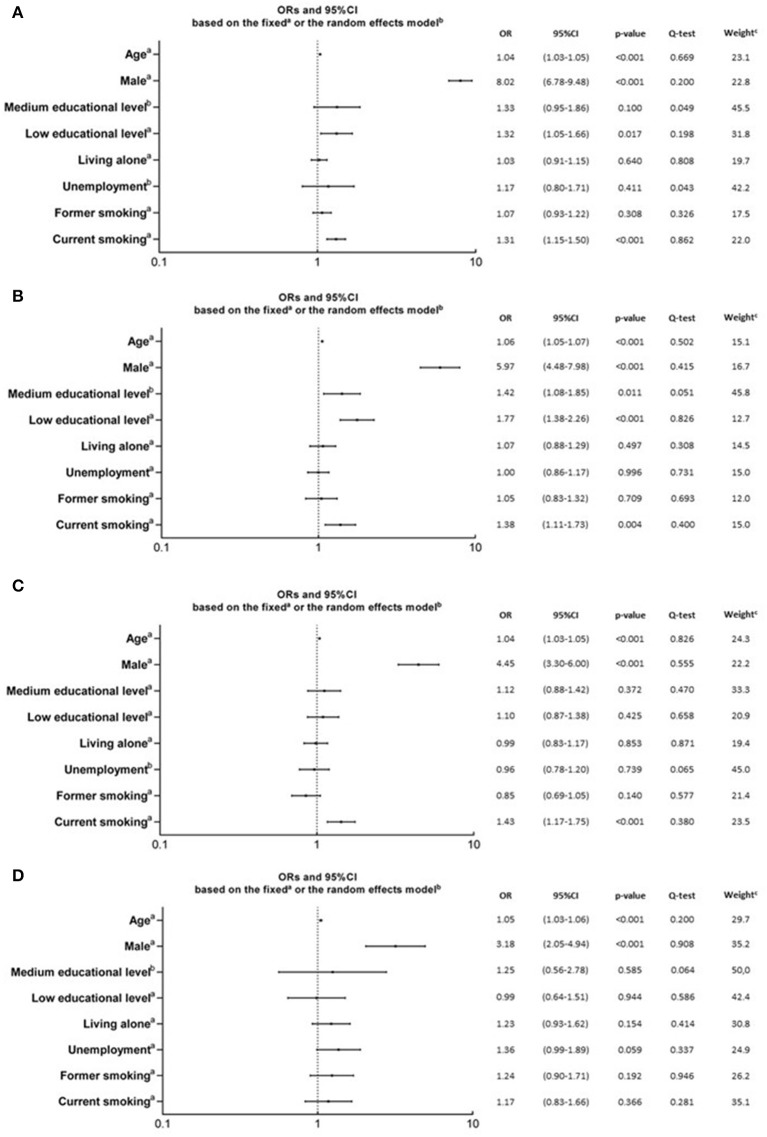
Pooled adjusted ORs for the association of behavioral and social factors with poor metabolic health, obtained from meta-analyses. These plots shows the pooled ORs in he overall population (**A**; *n* = 7,597), or stratified in normal weight (**B**; *n* = 3,643), overweight (**C**; *n* = 2,757), or obese (**D**; *n* = 1,197) subgroups. Pooled adjusted ORs were estimated through meta-analyses of adjusted ORs obtained from logistic regression analyses on each cohort. Meta-analyses were based on the fixed^a^ or the random^b^ effects model. Relative weight^c^ is related to adjusted ORs from the Kardiovize cohort, obtained through the fixed or the random effect model, and expressed as percentage. Metabolic health was defined according to the International Diabetes Federation (15).

To understand to what extent social and behavioral factors contributed to metabolic health, we meta-analyzed adjusted ORs obtained from logistic regression analyses on each cohort. The pooled adjusted ORs of the associations between social and behavioral factors and a metabolic unhealthy profile were calculated in the overall population and also stratified by BMI categories (Figure 2).

In the overall population, the main determinants of the metabolically unhealthy profile were increasing age, being male, low educational level, and current smoking (Figure 2A).

Among normal weight participants, the main determinants of the metabolically unhealthy profile were increasing age, being male, low-medium educational level, and current smoking (Figure 2B).

Among overweight participants, the main determinants of the metabolically unhealthy profile were increasing age, being male, and current smoking (Figure 2C).

Among obese participants, the main determinants of the metabolically unhealthy profile were only increasing age and being male (Figure 2D).

## Discussion

BMI is widely accepted as an obesity marker in population-based studies because, in the general population, metabolic health often declines as BMI increases. However, some obese individuals maintain metabolic health (2, 3). It is also increasingly clear that BMI is a rather poor indicator of body fat distribution and percentage at the individual level. For this reason, waist circumference and body fat mass remain among the most investigated obesity indicators in the research on metabolic health. In this scenario, our study evaluated the association between the above mentioned anthropometric measures and metabolic health in two urban population-based samples from Central Europe. In general, we confirmed that obesity traits were the main factors associated with poor metabolic health, but we also revealed some peculiar differences across BMI categories. In fact, waist circumference and body fat mass seemed to be associated with poor metabolic health among normal weight and overweight individuals from both cohorts. Conversely, the same indicators were not associated with the metabolic status among obese individuals. If it was true that participants could be considered metabolically unhealthy for reasons not related to obesity (type 1 diabetes, familiar dyslipidemia, essential hypertension etc.), the effects of other risk factors for poor metabolic health might partially explain this difference (9, 13).

Therefore, we aimed to provide the first comparison of determinants of metabolic health across BMI categories between two urban populations from Central-Eastern and Central-Western Europe. Interestingly, we demonstrated that increasing age, male gender, and low-medium educational level were the main social determinants of the metabolically unhealthy profile in both cohorts. Although educational level varies extremely between Czech and Swiss (17), it is considered the most important sociodemographic variable behind healthy choice, especially in terms of dietary habits (18). In fact, education might confer more knowledge about benefits and risks of dietary choices (19), and might also shape beliefs, attitudes, and behavior (20). In line, our study demonstrated that low educational level was associate with metabolically unhealthy profile among normal weight individuals from the Colaus cohort. Medium educational level, instead, was associated with unhealthy metabolic status among normal weight and obese individuals from the Kardiovize cohort.

With respect to behaviors, current smoking was one of the main determinants of metabolic health in normal weight individuals from the Colaus cohort and in overweight individuals from both cohort. Prevalence of current smokers and price of cigarettes are similar in Czech Republic and in Switzerland (21); however, when considering the GDP-PPP adjusted price of a pack of cigarettes and annual per adult cigarette consumption, Swiss consume almost the double the number of cigarettes than Czech people (21), both of domestic of foreign brands, which might help to explain the observed causative differences in epidemiological associations. Several observational studies demonstrated that current smoking was associated with abdominal obesity, but not with overall obesity, as in metabolically obese normal weight individuals (22, 23). For instance, a study enrolling 21,828 participants aged 45–79 from Norfolk (UK) found that, even after multivariable adjustment, abdominal obesity was highest among current smokers and lowest among never smokers (24). It has been suggested that smoking has some anti-estrogenic effect or may have an effect on the uptake and storage of triglyceride fatty acids, increasing fat mass (25).

To address to what extent clinical, social and behavioral determinants affected metabolic health, we finally meta-analyzed results obtained from the two cohorts. In general, a meta-analysis provides a summary of integrated results analyzed for their difference. The rationale behind our meta-analysis was to evaluate the associations of social and behavioral determinants with metabolic health among different subgroups of participants; to overcome limitations of small sample size, especially for some exposures; to feed new hypotheses and to inspire future studies with larger sample size.

In our study, increasing age and being male were certainly the main determinants of poor metabolic health, especially in the presence of obesity. In contrast, smoking status and low educational level were associated with poor metabolic health only in non-obese individuals. Overall, these results denoted that metabolic health in non-obese patients might be also associated with social and behavioral determinants, while metabolically unhealthy obesity appeared to be mostly attributed to aging and male gender. Moreover, it has not yet been fully elucidated whether metabolically unhealthy obese individuals are genetically or epigenetically predisposed (11), or whether metabolically healthy obesity represents a transitory state to metabolically unhealthy conditions (26).

Our study has several strengths. Firstly, it was based in large samples randomly selected from the urban population of Brno (Czech Republic) and Lausanne (Switzerland). Secondly, cardio-metabolic parameters and anthropometric measures were assessed using standard and validated protocols. Thirdly, the availability of two similar datasets allowed us to exploit the same statistical approach, instead of relying on previously published data. Yet, the comparison of two datasets of information that have been collected by different but comparable methods requires caution. In our study, however, we used the same inclusion criteria for both cohorts, and we analyzed the variables that were collected by comparable methods (e.g., structured questionnaires for sociodemographic and behavioral data, and standardized and validated protocols for clinical data). Finally, the majority of the results are robust, as they have been confirmed after adjusting for several socio-demographic and behavioral factors and were found for both cohorts using logistic regression analysis.

Our study also has some limitations. Firstly, its cross-sectional design precludes assessing causality. Taking into account the number of years of exposure to obesity will be of utmost importance in future longitudinal studies about metabolic health in human obesity. Secondly, the effect of unmeasured socio-demographic factors (e.g., income, food security, food access), behaviors (i.e., dietary habits, sleep deprivation, physical activity), physiological condition (e.g., menopause status) and comorbidities cannot be completely excluded. Moreover, the inclusion of retired people in the unemployed groups may introduce potential bias, which should be considered when interpreting our findings. Finally, it was not possible to have a more detailed assessment of adiposity such as visceral fat area, active body mass-muscle mass, and epicardial fat between Czech and Swiss populations.

In conclusion, our findings suggested that body fat mass and its distribution—expressed in terms percentage and waist circumference—were associated with metabolic health only among non-obese individuals. Our study, to the best of our knowledge, is also the first providing a comparison of social and behavioral determinants of metabolic health in two Central European cohorts. Interestingly, it seemed reasonable that age and gender affected metabolic profile in each BMI category, while modifiable factors—such as educational level and smoking habits—appeared crucial only in non-obese individuals. In line, public health strategies against obesity and related comorbidities should aim to improve social conditions and to promote healthy lifestyles before the progression of metabolic disorders.

## Data Availability Statement

The datasets generated for this study are available on request to the corresponding author.

## Ethics Statement

The baseline study protocol of the Kardiovize Brno 2030 study was approved by the Ethics Committee of St Anne's University Hospital, Brno, Czech Republic (reference 2 G/2012). The institutional Ethics Committee of the University of Lausanne, which afterwards became the Ethics Commission of Canton Vaud (www.cer-vd.ch), approved the baseline CoLaus study (reference 16/03, decisions of 13th January and 10th February 2003), and the approval was renewed for each follow-up. Both studies were performed in agreement with the Helsinki declaration and its former amendments. The patients/participants provided their written informed consent to participate in this study.

## Author Contributions

MV and PM-V designed the study, obtained the data, and wrote part of the manuscript. SK and AM ran the statistical analysis and wrote part of the manuscript. JM-I and FL-J revised the manuscript critically for important intellectual content. All authors have given final approval of the version to be published.

### Conflict of Interest

The authors declare that the research was conducted in the absence of any commercial or financial relationships that could be construed as a potential conflict of interest.
