# Supplementary material for: Determinants of Metabolic Health Across Body Mass Index Categories in Central Europe: A Comparison Between Swiss and Czech Populations
Source: Front Public Health. 2020 Apr 8;8:108. doi: 10.3389/fpubh.2020.00108 (PMC7156604; doi:10.3389/fpubh.2020.00108)
Supplement: Supplementary file 1 [file Data_Sheet_1.docx]

Supplementary Material

**Supplementary Table 1.** Characteristics of participants according to metabolic status

| Characteristics ^a^ | Kardiovize (n=1852) | | | Colaus (n=5745) | | |
| --- | --- | --- | --- | --- | --- | --- |
|  | **Metabolically Healthy** | **Metabolically Unhealthy** | **p-value** | **Metabolically Healthy** | **Metabolically Unhealthy** | **p-value** |
| Sample size | 1336 | 516 |  | 3453 | 2292 |  |
| Men | 41.1% (549) | 57.4% (296) | **< 0.001** | 37.7% (1303) | 61.4% (1408) | **< 0.001** |
| Age, years | 45.0 (18.0) | 55.0 (14.0) | **< 0.001** | 48.2 (16.0) | 56.9 (15.0) | **< 0.001** |
| Marital status |  |  | 0.055 |  |  | **0.003** |
| Living alone | 39.3% (523) | 34.4% (177) |  | 42.4% (1463) | 38.4% (880) |  |
| Living in couple | 60.7% (809) | 65.6% (337) |  | 57.6% (1989) | 61.6% (1411) |  |
| Educational status |  |  | **< 0.001** |  |  | **< 0.001** |
| High | 46.6% (621) | 30.5% (157) |  | 22.1% (764) | 14.9% (340) |  |
| Medium | 36.4% (485) | 43.5% (224) |  | 25.8% (891) | 21.3% (488) |  |
| Low | 17.0% (227) | 26.0% (134) |  | 52.0% (1796) | 63.8% (1461) |  |
| Employment status |  |  | **< 0.001** |  |  | **< 0.001** |
| Unemployed | 14.3% (185) | 25.2% (125) |  | 25.8% (891) | 37.5% (858) |  |
| Employed | 85.7% (1109) | 74.8% (372) |  | 74.2% (2561) | 62.5% (1433) |  |
| Smoking status |  |  | **0.004** |  |  | **< 0.001** |
| Never | 53.5% (714) | 45.2% (233) |  | 43.4% (1498) | 37.4% (857) |  |
| Former | 21.1% (281) | 23.1% (119) |  | 29.9% (1032) | 35.3% (808) |  |
| Current | 25.4% (339) | 31.7% (163) |  | 26.7% (922) | 27.3% (626) |  |
| Abdominal obesity | 21.7% (289) | 62.9% (324) | **< 0.001** | 18.7% (645) | 45.9% (1053) | **< 0.001** |
| BMI, kg/m^2^ | 24.2 (4.9) | 28.7 (5.9) | **< 0.001** | 23.9 (4.6) | 27.5 (5.5) | **< 0.001** |
| Waist circumference, cm | 85.0 (17.0) | 101.0 (16.0) | **< 0.001** | 84.0 (16.0) | 96.0 (16.0) | **< 0.001** |
| Body fat mass, % | 23.7 (12.6) | 28.8 (14.5) | **< 0.001** | 28.0 (12.1) | 29.0 (14.0) | **< 0.001** |
| SBP, mmHg | 114.5 (16.5) | 128.5 (19.0) | **< 0.001** | 120.0 (17.0) | 136.3 (20.0) | **< 0.001** |
| DBP, mmHg | 77.5 (12.0) | 85.5 (11.4) | **< 0.001** | 75.5 (12.0) | 84.5 (12.5) | **< 0.001** |
| Glucose, mmol/L | 4.8 (0.6) | 5.4 (1.0) | **< 0.001** | 5.2 (0.5) | 5.8 (0.7) | **< 0.001** |
| Total cholesterol, mmol/L | 5.1 (1.3) | 5.3 (1.4) | **0.001** | 5.4 (1.3) | 5.8 (1.4) | **< 0.001** |
| Triglycerides, mmol/L | 0.9 (0.6) | 1.8 (0.9) | **< 0.001** | 0.9 (0.5) | 1.7 (1.1) | **< 0.001** |
| HDL cholesterol, mmol/L | 1.6 (0.5) | 1.2 (0.4) | **< 0.001** | 1.7 (0.5) | 1.4 (0.5) | **< 0.001** |
| LDL cholesterol, mmol/L | 3.0 (1.2) | 3.2 (1.3) | **0.015** | 3.2 (1.2) | 3.5 (1.2) | **< 0.001** |
| Diagnosis of type 1 diabetes | 0.2% (3) | 1.7% (9) | **< 0.001** | 0.1% (2) | 0.4% (9) | **0.004** |
| Diagnosis of type 2 diabetes | 0.4% (5) | 10.3% (53) | **< 0.001** | 0.6% (19) | 8.7% (199) | **< 0.001** |
| Oral antidiabetic treatment | 0.2% (3) | 8.7% (45) | **< 0.001** | 0.3% (11) | 6.6% (151) | **< 0.001** |
| Insulin treatment | 0.2% (3) | 2.5% (13) | **< 0.001** | 0.0% (1) | 1.4% (32) | **< 0.001** |
| Diagnosis of hypertension | 18.5% (245) | 62.6% (320) | **< 0.001** | 13.0% (449) | 43.7% (1001) | **< 0.001** |
| Antihypertensive drug treatment | 9.1% (121) | 50.6% (261) | **< 0.001** | 6.4% (223) | 31.4% (720) | **< 0.001** |
| Diagnosis of high cholesterol | 20.8% (270) | 50.1% (250) | **< 0.001** | 12.2% (420) | 37.2% (852) | **< 0.001** |
| Hypolipidemic drug treatment | 1.3% (17) | 22.3% (115) | **< 0.001** | 2.1% (71) | 22.7% (520) | **< 0.001** |

BMI, body mass index; SBP, systolic blood pressure; DBP, diastolic blood pressure; HDL, high-density lipoprotein; LDL, low-density lipoprotein.^a^ Results are reported as median (interquartile range) or percentage (frequency). Statistical analysis using Chi-square test for bivariate or categorical variable, and Mann-Whitney U test for continuous variables.

**Supplementary Table 2**. Characteristics of normal weight participants according to metabolic status

| Characteristics ^a^ | Kardiovize | | | CoLaus | | |
| --- | --- | --- | --- | --- | --- | --- |
|  | **Metabolically Healthy** | **Metabolically Unhealthy** | **p-value** | **Metabolically Healthy** | **Metabolically Unhealthy** | **p-value** |
| Sample size | 797 | 90 |  | 2163 | 593 |  |
| Men | 34.8% | 44.4% | 0.069 | 32.6% | 56.2% | < 0.001 |
| Age, years | 42.0 (16.0) | 55.0 (17.0) | < 0.001 | 47.4 (16.0) | 56.5 (16.0) | < 0.001 |
| Marital status |  |  | 0.858 |  |  | 0.106 |
| Living alone | 41.7% | 42.7% |  | 44.5% | 40.8% |  |
| Living in couple | 58.3% | 57.3% |  | 55.5% | 59.2% |  |
| Educational level |  |  | < 0.001 |  |  | < 0.001 |
| High | 51.1% | 26.7% |  | 26.1% | 17.5% |  |
| Medium | 35.3% | 52.2% |  | 27.9% | 22.9% |  |
| Low | 13.6% | 21.1% |  | 45.9% | 59.5% |  |
| Employment status |  |  | 0.030 |  |  | < 0.001 |
| Unemployed | 14.2% | 23.0% |  | 22.9% | 35.6% |  |
| Employed | 85.8% | 77.0% |  | 77.1% | 64.4% |  |
| Smoking status |  |  | 0.187 |  |  | 0.002 |
| Never | 56.2% | 47.8% |  | 42.4% | 34.6% |  |
| Former | 18.4% | 17.8% |  | 28.5% | 30.7% |  |
| Current | 25.7% | 34.4% |  | 29.1% | 34.7% |  |
| Abdominal obesity | 1.8% | 10.1% | < 0.001 | 3.7% | 6.1% | 0.011 |
| BMI, kg/m^2^ | 22.3 (2.9) | 23.5 (2.3) | < 0.001 | 22.4 (2.7) | 23.3 (2.3) | < 0.001 |
| Waist circumference, cm | 78.5 (11.0) | 85.0 (11.0) | < 0.001 | 78.0 (12.0) | 85.0 (12.0) | < 0.001 |
| Body fat mass, % | 21.3 (10.5) | 23.5 (9.9) | 0.003 | 26.0 (11.7) | 24.7 (12.5) | 0.627 |
| SBP, mm Hg | 112.0 (15.5) | 128.5 (17.3) | < 0.001 | 118.0 (17.0) | 135.0 (19.5) | < 0.001 |
| DBP, mm Hg | 76.0 (11.5) | 84.3 (11.9) | < 0.001 | 74.0 (11.5) | 83.0 (13.0) | < 0.001 |
| Glucose. mmol/L | 4.7 (0.6) | 5.2 (0.9) | < 0.001 | 5.1 (0.6) | 5.7 (0.6) | < 0.001 |
| Total cholesterol. mmol/L | 5.0 (1.3) | 5.3 (1.5) | 0.014 | 5.3 (1.3) | 5.7 (1.4) | < 0.001 |
| Triglycerides. mmol/L | 0.8 (0.5) | 1.7 (1.1) | < 0.001 | 0.9 (0.4) | 1.5 (1.1) | < 0.001 |
| HDL cholesterol. mmol/L | 1.7 (0.5) | 1.3 (0.5) | < 0.001 | 1.8 (0.6) | 1.6 (0.6) | < 0.001 |
| LDL cholesterol. mmol/L | 2. 9 (1.1) | 3.2 (1.6) | 0.056 | 3.1 (1.2) | 3.3 (1.3) | < 0.001 |
| Diagnosis of type 1 diabetes | 0.4% | 6.7% | < 0.001 | 0.1% | 0.5% | 0.036 |
| Diagnosis of type 2 diabetes | 0.4% | 4.4% | < 0.001 | 0.4% | 4.4% | < 0.001 |
| Oral antidiabetic treatment | 0.1% | 1.1% | 0.062 | 0.2% | 2.9% | < 0.001 |
| Insulin treatment | 0.4% | 7.8% | < 0.001 | 0.0% | 1.7% | < 0.001 |
| Diagnosis of hypertension | 13.4% | 44.9% | < 0.001 | 9.3% | 32.2% | < 0.001 |
| Antihypertensive drug treatment | 6.3% | 35.6% | < 0.001 | 3.8% | 22.3% | < 0.001 |
| Diagnosis of high cholesterol | 17.3% | 46.7% | < 0.001 | 10.5% | 31.0% | < 0.001 |
| Hypolipidemic drug treatment | 0.8% | 21.1% | < 0.001 | 1.9% | 21.4% | < 0.001 |

BMI, body mass index; SBP, systolic blood pressure; DBP, diastolic blood pressure; HDL, high-density lipoprotein; LDL, low-density lipoprotein. ^a^ Results are reported as median (interquartile range) or percentage. Statistical analysis using Chi-square test for bivariate or categorical variable, and Mann-Whitney U test for continuous variables.

**Supplementary Table 3**. Characteristics of overweight participants according to metabolic status

| Characteristics ^a^ | Kardiovize | | | CoLaus | | |
| --- | --- | --- | --- | --- | --- | --- |
|  | **Metabolically Healthy** | **Metabolically Unhealthy** | **p-value** | **Metabolically Healthy** | **Metabolically Unhealthy** | **p-value** |
| Sample size | 401 | 230 |  | 1044 | 1082 |  |
| Men | 53.9% | 68.7% | < 0.001 | 48.6% | 67.8% | < 0.001 |
| Age, years | 47.0 (18.0) | 53.5 (15.0) | < 0.001 | 50.0 (18.0) | 56.70 (16.0) | < 0.001 |
| Marital status |  |  | 0.306 |  |  | 0.234 |
| Living alone | 34.4% | 30.4% |  | 39.9% | 37.4% |  |
| Living in couple | 65.6% | 69.6% |  | 60.1% | 62.6% |  |
| Educational level |  |  | 0.055 |  |  | 0.999 |
| High | 42.3% | 35.8% |  | 16.7% | 16.8% |  |
| Medium | 39.0% | 37.6% |  | 22.6% | 22.6% |  |
| Low | 18.8% | 26.6% |  | 60.7% | 60.6% |  |
| Employment status |  |  | 0.006 |  |  | 0.063 |
| Unemployed | 13.2% | 21.7% |  | 30.0% | 33.8% |  |
| Employed | 86.8% | 78.3% |  | 70.0% | 66.2% |  |
| Smoking status |  |  | 0.020 |  |  | 0.002 |
| Never | 49.6% | 42.8% |  | 43.7% | 36.4% |  |
| Former | 26.7% | 23.1% |  | 32.7% | 37.0% |  |
| Current | 23.7% | 34.1% |  | 23.6% | 26.6% |  |
| Abdominal obesity | 36.6% | 52.2% | < 0.001 | 33.1% | 41.1% | < 0.001 |
| BMI, kg/m^2^ | 26.7 (2.1) | 27.7 (2.5) | < 0.001 | 26.7 (2.2) | 27.4 (2.3) | < 0.001 |
| Waist circumference, cm | 92.0 (11.0) | 99.0 (9.0) | < 0.001 | 91.0 (12.0) | 96.0 (10.0) | < 0.001 |
| Body fat mass, % | 27.0 (13.5) | 26.4 (11.2) | 0.786 | 30.0 (14.3) | 27.2 (12.4) | 0.062 |
| SBP, mm Hg | 116.0 (15.3) | 128.5 (19.8) | < 0.001 | 122.5 (17.0) | 135.5 (19.0) | < 0.001 |
| DBP, mm Hg | 78.5 (11.8) | 86.0 (12.1) | < 0.001 | 77.0 (11.0) | 84.8 (12.5) | < 0.001 |
| Glucose. mmol/L | 4.9 (0.6) | 5.3 (0.9) | < 0.001 | 5.2 (0.5) | 5.8 (0.6) | < 0.001 |
| Total cholesterol. mmol/L | 5.2 (1.3) | 5.4 (1.4) | 0.161 | 5.6 (1.3) | 5.9 (1.5) | < 0.001 |
| Triglycerides. mmol/L | 1.1 (0.6) | 1.9 (1.0) | < 0.001 | 1.0 (0.5) | 1.7 (1.1) | < 0.001 |
| HDL cholesterol. mmol/L | 1.5 (0.5) | 1.3 (0.4) | < 0.001 | 1.6 (0.5) | 1.4 (0.4) | < 0.001 |
| LDL cholesterol. mmol/L | 3.2 (1.2) | 3.3 (1.3) | 0.977 | 3.4 (1.1) | 3.6 (1.2) | 0.002 |
| Diagnosis of type 1 diabetes | 0.0% | 1.3% | 0.022 | 0.0% | 0.4% | 0.049 |
| Diagnosis of type 2 diabetes | 0.0% | 9.6% | < 0.001 | 0.6% | 6.2% | < 0.001 |
| Oral antidiabetic treatment | 0.2% | 9.1% | < 0.001 | 0.4% | 4.5% | < 0.001 |
| Insulin treatment | 0.0% | 1.3% | 0.022 | 0.0% | 0.7% | 0.005 |
| Diagnosis of hypertension | 21.5% | 55.1% | < 0.001 | 17.0% | 42.1% | < 0.001 |
| Antihypertensive drug treatment | 10.2% | 41.3% | < 0.001 | 9.0% | 29.3% | < 0.001 |
| Diagnosis of high cholesterol | 26.7% | 53.4% | < 0.001 | 14.9% | 38.2% | < 0.001 |
| Hypolipidemic drug treatment | 0.8% | 21.1% | < 0.001 | 1.9% | 21.4% | < 0.001 |

BMI, body mass index; SBP, systolic blood pressure; DBP, diastolic blood pressure; HDL, high-density lipoprotein; LDL, low-density lipoprotein. ^a^ Results are reported as median (interquartile range) or percentage. Statistical analysis using Chi-square test for bivariate or categorical variable, and Mann-Whitney U test for continuous variables.

**Supplementary Table 4**. Characteristics of obese participants according to metabolic status

| Characteristics ^a^ | Kardiovize | | | CoLaus | | |
| --- | --- | --- | --- | --- | --- | --- |
|  | **Metabolically Healthy** | **Metabolically Unhealthy** | **p-value** | **Metabolically Healthy** | **Metabolically Unhealthy** | **p-value** |
| Sample size | 138 | 196 |  | 246 | 617 |  |
| Men | 40.6% | 50.0% | 0.089 | 37.0% | 55.3% | < 0.001 |
| Age, years | 51.0 (17.0) | 56.0 (12.0) | 0.001 | 48.5 (17.0) | 57.6 (14.0) | < 0.001 |
| Marital status |  |  | 0.485 |  |  | 0.299 |
| Living alone | 39.1% | 35.4% |  | 34.1% | 37.9% |  |
| Living in couple | 60.9% | 64.6% |  | 65.9% | 62.1% |  |
| Educational level |  |  | 0.098 |  |  | 0.453 |
| High | 33.3% | 26.0% |  | 9.8% | 8.9% |  |
| Medium | 34.8% | 46.4% |  | 20.8% | 17.5% |  |
| Low | 31.9% | 27.6% |  | 69.4% | 73.5% |  |
| Employment status |  |  | 0.015 |  |  | 0.001 |
| Unemployed | 18.2% | 30.2% |  | 33.7% | 45.7% |  |
| Employed | 81.8% | 69.8% |  | 66.3% | 54.3% |  |
| Smoking status |  |  | 0.537 |  |  | 0.061 |
| Never | 50.7% | 46.9% |  | 50.8% | 42.0% |  |
| Former | 20.3% | 25.5% |  | 30.5% | 36.6% |  |
| Current | 29.1% | 27.6% |  | 18.7% | 21.4% |  |
| Abdominal obesity | 93.5% | 99.5% | 0.002 | 89.4% | 92.7% | 0.114 |
| BMI, kg/m^2^ | 32.5 (4.0) | 33.0 (4.7) | 0.184 | 32.1 (3.3) | 32.8 (4.1) | < 0.001 |
| Waist circumference, cm | 104.5 (12.0) | 110.0 (14.0) | < 0.001 | 102.0 (14.0) | 108.0 (13.0) | < 0.001 |
| Body fat mass, % | 38.6 (13.8) | 38.1 (13.7) | 0.812 | 40.0 (15.0) | 36.0 (14.8) | 0.046 |
| SBP, mm Hg | 124.5 (17.8) | 128.5 (19.8) | < 0.001 | 125.0 (19.6) | 138.0 (21.3) | < 0.001 |
| DBP, mm Hg | 82.5 (10.0) | 84.5 (11.0) | 0.052 | 80.8 (12.6) | 86.0 (12.5) | < 0.001 |
| Glucose. mmol/L | 5.0 (0.6) | 5.6 (1.1) | < 0.001 | 5.2 (0.5) | 6.0 (1.1) | < 0.001 |
| Total cholesterol. mmol/L | 5.3 (1.3) | 5.2 (1.3) | 0.114 | 5.5 (1.4) | 5.7 (1.4) | 0.010 |
| Triglycerides. mmol/L | 1.1 (0.6) | 1.7 (0.9) | < 0.001 | 1.2 (0.5) | 1.8 (1.3) | < 0.001 |
| HDL cholesterol. mmol/L | 1.4 (0.4) | 1.2 (0.3) | < 0.001 | 1.6 (0.5) | 1.3 (0.4) | < 0.001 |
| LDL cholesterol. mmol/L | 3.4 (1.4) | 3.1 (1.2) | 0.013 | 3.3 (1.2) | 3.4 (1.1) | 0.240 |
| Diagnosis of type 1 diabetes | 0.0% | 0.0% | - | 0.0% | 0.3% | 0.371 |
| Diagnosis of type 2 diabetes | 1.4% | 13.8% | < 0.001 | 2.0% | 17.2% | < 0.001 |
| Oral antidiabetic treatment | 0.7% | 11.7% | < 0.001 | 1.2% | 13.8% | < 0.001 |
| Insulin treatment | 0.0% | 1.5% | 0.144 | 0.0% | 2.3% | 0.017 |
| Diagnosis of hypertension | 39.1% | 79.5% | < 0.001 | 28.0% | 57.4% | < 0.001 |
| Antihypertensive drug treatment | 21.7% | 68.4% | < 0.001 | 18.7% | 43.9% | < 0.001 |
| Diagnosis of high cholesterol | 24.3% | 47.9% | < 0.001 | 14.6% | 41.3% | < 0.001 |
| Hypolipidemic drug treatment | 3.6% | 21.9% | < 0.001 | 1.6% | 24.6% | < 0.001 |

BMI, body mass index; SBP, systolic blood pressure; DBP, diastolic blood pressure; HDL, high-density lipoprotein; LDL, low-density lipoprotein. ^a^ Results are reported as median (interquartile range) or percentage. Statistical analysis using Chi-square test for bivariate or categorical variable, and Mann-Whitney U test for continuous variables.
